# Genomic Diversity of Avocado in the Morogoro Region and Southern Highlands of Tanzania

**DOI:** 10.3390/ijms27073083

**Published:** 2026-03-28

**Authors:** Andrés J. Cortés, Juma M. Hussein, Ibrahim Juma

**Affiliations:** 1 Department of Plant Breeding, Swedish University of Agricultural Sciences, 23436 Lomma, Sweden; 2 Department of Molecular Biology and Biotechnology, University of Dar es Salaam, P.O. Box 35179 Dar es Salaam, Tanzania; jmhussein@udsm.ac.tz; 3 Department of Food Science and Technology, University of Dar es Salaam, P.O. Box 35134 Dar es Salaam, Tanzania; vuga.ibrahim@udsm.ac.tz

**Keywords:** *Persea americana* L., *in situ* conservation, East Africa, ‘plus trees’, low-coverage whole-genome resequencing (lcWGS)

## Abstract

Avocado (*Persea americana* Mill.) is one of the most widely consumed fruit tree crops worldwide, with cultivation expanding rapidly beyond its Mesoamerican and northwest South America center of origin. In emerging secondary diversity centers such as East Africa, farmers have long propagated seedling naturalized populations that may hold valuable reservoirs of genetic diversity, yet these resources remain underexplored. To help fill this gap, this study developed the first genomic resources for avocados in Tanzania, where avocado has a long history of introduction and diversification dating to the first Arab incursions and Catholic missionary missions. Low-coverage whole-genome resequencing (lcWGS) data were obtained from 95 trees sampled in Tanzania across the low- to mid-altitude Morogoro region (*n* = 25) and the Southern Highlands—i.e., the Iringa (*n* = 20), Mbeya (*n* = 30) and Ruvuma (*n* = 20) regions. In order to guide racial assignation, sequences were merged with NCBI-available lcWGS data from 205 avocado trees, including 42 commercial varieties, with reported ancestry. Population stratification as inferred via maximum likelihood phylogenetic inference, genetic principal component analysis, and ADMIXTURE unsupervised clustering suggested that the sampled Tanzanian avocado trees were genetically closer to the West Indian race and more distant from the northwest South American Caribbean and Andean groups. Additionally, while the trees from the low- to mid-altitude region of Morogoro were almost exclusively West Indian type, some trees from the Southern Highlands aligned more closely with West Indian × Guatemalan and West Indian × Mexican hybrids. These trends were equally supported by a subset of 10,460 high-coverage (10×) SNP markers. Together these findings clarify the dynamics of avocado diversification in a secondary center in East Africa, spanning recent introductions from a single Mesoamerican race, adaptation to a wide range of locally geographic conditions, and farmer-driven selection matching local tribal preferences. Characterizing these locally adapted resources is key for identifying underrepresented yet promising provenances, developing resilient and sustainable horticultural production systems, and safeguarding the species’ global genetic heritage.

## 1. Introduction

Avocado (*Persea americana* Mill.) is among the most economically important and nutritionally valuable fruit tree crops worldwide [1], with global demand steadily increasing due to its high content of monounsaturated fatty acids, vitamins, and bioactive compounds [2,3,4,5,6]. Native to Mesoamerica and northwest South America [7,8], avocado diversity has historically been structured in three classical botanical races [7,9,10,11]—Mexican, Guatemalan, and West Indian—the first two adapted to high-altitude cooler environments, the later more associated with warmer lowlands [12,13]. More recently, genomic evidence has further distinguished unique, Pleistocene-dating, Caribbean and Andean linages in northwest South America [14,15], deepening our understanding of avocado’s evolutionary history.

Beyond its primary center of origin, avocado cultivation has expanded extensively into secondary regions across the tropics and subtropics. In East Africa, and particularly in Tanzania, avocado has a long yet fragmented history of introduction, beginning with early trade routes associated with Arab incursions and later reinforced by Catholic missionary settlers and colonial agricultural programs [16]. Over generations, farmer-mediated seed propagation, local trading networks, and adaptation to heterogeneous micro-agroecological conditions have uniquely shaped these naturalized populations [17]. Contrasting environments such as the low- to mid-altitude region of Morogoro and the Southern Highlands—including Iringa, Mbeya and Ruvuma regions—may therefore harbor unique genetic diversity and locally adaptive alleles relevant for tolerance to abiotic stresses including drought, heat, cooler temperatures and variable rainfall regimes.

Despite avocado’s growing economic importance in East Africa, genomic resources for Tanzanian germplasm are absent. Most population genomic studies have focused on materials from Mesoamerica [18,19,20,21], northwest South America [22,23,24,25], secondary centers in Europe [26,27] and South Africa [28], or commercial cultivars [29] like ‘Hass’ [30] and ’Gwen’ [31], leaving secondary diversification in East Africa poorly characterized. Thus, the racial ancestry, degree of introgression, and internal population stratification of Tanzanian avocado populations are largely unknown. It remains unclear whether these naturalized trees derive predominantly from a Mesoamerican race, represent admixed lineages introduced through multiple routes, or constitute emerging, locally differentiated genepools shaped by environmental selection and farmer preferences.

This knowledge gap limits the conservation and utilization of Tanzanian germplasm, jeopardizing efforts to leverage local diversity as novel avocado varieties or within ongoing rootstock breeding programs. In particular, contrasting agroecological conditions between the warm, low- to mid-altitude Morogoro region, and the cooler, higher-altitude Southern Highlands offer a natural gradient to explore how introduction history, gene flow, and local adaptation have interplayed to structure genomic diversity in a secondary diversity center. Afterall, in perennial fruit trees crops such as avocado—characterized by allogamy, long generation times and incipient domestication [32,33,34]—*in situ* farmer-conserved seedling populations may harbor vital reservoirs of genetic novelty [35,36].

Integrating low-coverage whole-genome resequencing (lcWGS) [37], multivariate population genomics, and admixture modeling would enable the bridging of this gap by offering fine-scale reconstructions of ancestry, introgression, and divergence patterns, even in recently established secondary diversity centers. These last-generation genomic tools can further inform marker-assisted selection, genomic prediction, and pre-breeding pipelines aimed at enhancing avocado productivity, resilience and sustainability. Hence, the study goals were to (*i*) generate the first genome-wide SNP dataset for Tanzanian avocado germplasm using lcWGS, (*ii*) reconstruct the racial ancestry of trees from Morogoro and the Southern Highlands by integrating racial controls’ lcWGS, and (*iii*) assess the population stratification and extent of introgression in avocados from Tanzania.

To help fill the research gap, this study investigated which classical Mesoamerican avocado races or newly defined northwest South American genetic groups are Tanzanian populations genetically closely related to, and whether the agroecologically distinct regions of low- to mid-altitude Morogoro and the Southern Highlands harbor genomically differentiated or admixed avocado lineages. We hypothesized that (*i*) Tanzanian avocado trees would cluster predominantly with the West Indian race, consistent with adaptation to tropical climates, (*ii*) trees from the Southern Highlands would exhibit greater introgression from Guatemalan and/or Mexican races due to allelic filtering for adaptation to cooler, higher-elevation environments, and (*iii*) regional-specific genomic signatures would reflect emerging differentiation within this secondary center of diversity. Revealing the genomic diversity of Tanzanian avocados would assist conservation and molecular breeding efforts targeting resilient and sustainable horticultural production under changing climatic conditions in an understudied yet promising hub in East Africa.

## 2. Results

With the aim of reconstructing the population stratification and racial ancestry of avocado germplasm from the low- to mid-altitude Morogoro region and the Southern Highlands of Tanzania, low-coverage whole-genome resequencing (lcWGS) was carried out on 95 avocado trees, and the sequences obtained were merged with the NCBI-available lcWGS data from 205 avocados with reported racial ancestry [14]. This full lcWGS-based SNP panel was analyzed through phylogenetic, multivariate and admixture tools. lcWGS genotyping placed the Tanzania avocados closer to the West Indian race, with introgression from the Guatemalan and Mexican races for some trees from the Southern Highlands. The ancestry, local divergence and introgression patterns of the analyzed Tanzanian germplasm highlights prospects for diversifying breeding by eventually testing novel provenances in East Africa, particularly from underrepresented racial groups.

### 2.1. lcWGS-Based SNP Markers

Low-coverage whole-genome resequencing (lcWGS) of 95 Tanzanian avocado trees (Table S1), spanning the low- to mid-altitude Morogoro region and the Southern Highlands (i.e., Iringa, Mbeya and Ruvuma regions), yielded an average of 30,648,133 reads and 4,627,868,040 bp per sample with a mean G:C content of 40.04%, Q20 of 97.55% and Q30 of 93.46% (Table S2). An average of 29,628,649 (96.67%) reads passed quality filters at a mean depth of 4.83× (Table S3), 29,439,508 (99.35%) of which mapped against the avocado var. Hass reference genome [30] with an average duplicate rate of 13.11% (Table S4). An average of 81.72% mapped reads per sample had a minimum depth ≥ 1×, 46.28% ≥ 4×, 5.28% ≥ 10×, and 1.15% ≥ 20× (Table S5).

After merging this dataset and the 205 controls with reported racial ancestry, SNP calling yielded an average transition/transversion (Ts/Tv) ratio of 2.19 (Table S6). A total of 40,994,428 and 14,748,136 SNPs mapped to non-coding inter-genic and intronic regions, respectively, while 1,868,065 were exonic, 745,468 synonymous and 1,082,915 non-synonymous. Additionally, 34,027 and 4162 SNPs caused a gain or a loss of a stop codon, respectively, and 12,019 altered the splicing pattern. Finally, 2,882,670 SNPs fell in the 1 kb window upstream of the transcription start site, while 2,415,218 SNPs fell in the 1 kb window downstream of the transcription termination site.

Meanwhile, more stringent marker filtering (i.e., sequencing depth of at least 10× per sample per position, missing data per variant not exceeding 20%, and *maf* ≥ 5%) led to a smaller dataset of 10,460 high-coverage SNP markers, explored for corroborative phylogenetic inference and unsupervised multivariate clustering analyses, as well as a resource for downstream applications by the community.

### 2.2. Phylogenetic, Multivariate and Admixture Reconstructions

**Figure 1:**
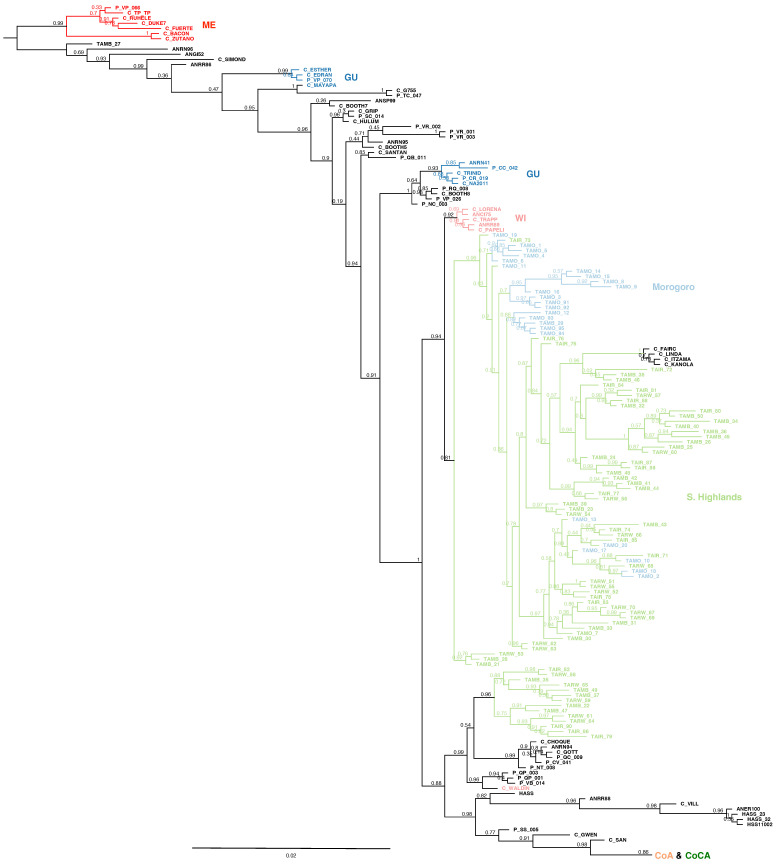
Rooted phylogenetic inference in 95 Tanzanian avocado trees from the low- to mid-altitude Morogoro region (light blue) and the Southern Highlands (i.e., Iringa, Mbeya and Ruvuma regions) (light green), together with 205 NCBI-available racial controls [14], all genotyped with high-coverage lcWGS-derived SNPs (full set in Figure S1). Controls colored as: Mexican (ME) race in dark red, Guatemalan (GU) race in dark blue, and West Indian (WI) in light red. The last branch contains the northwest South American Andean (CoA) and Caribbean (CoCA) clades (Figure S2).

Phylogenetic inference grouped the majority of Tanzanian samples as a sister clade of the West Indian race (Figure 1), separated from the Mexican and Guatemalan racial controls, as well as the Andean and Caribbean groups from northwest South America.

**Figure 2:**
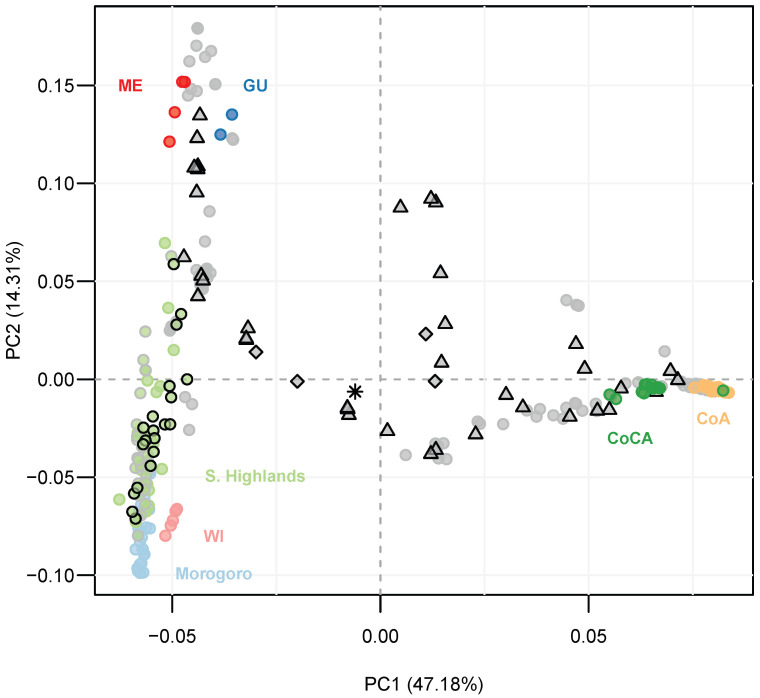
Principal component analysis (PCA) displaying unsupervised genetic clustering of 95 Tanzanian avocado trees from the low- to mid-altitude Morogoro region (light blue) and the Southern Highlands (light green), with 205 NCBI-available racial controls [14]—all genotyped with lcWGS-derived SNPs. Trees from the Southern Highlands in Tanzania are colored as follows: light green dots with green edge are from the Iringa region, while those with gray and black edges are from the Mbeya and Ruvuma regions, respectively. Racial controls are labeled and colored top down from left to right, as follows: dark red for the Mexican (ME) race, dark blue for the Guatemalan (GU) race, light red for the West Indian (WI) race, and dark green and light orange for the Caribbean (CoCA) and Andean (CoA) groups from northwest South America. Gray dots mark double hybrids between these last five racial control groups, while triangles, diamonds and the asterisk, respectively, mark the triple, tetra and penta hybrids. The detailed assignation of the admixed genotypes (i.e., gray dots, triangles and diamonds) to the corresponding putative parental racial groups is detailed in the next figure. Principal component (PC)-explained variance is given in the axes’ labels.

On the other hand, principal component analysis (PCA) recovered seven groups (Figure 2, Table S7), with PC1 and PC2 accounting for 47.18% and 14.31% of variance, respectively. Data projection towards the main component positioned the Tanzanian samples from the low- to mid-altitude Morogoro region and the Southern Highlands closer to the Mesoamerican races (i.e., Mexican ME, Guatemalan GU, and West Indian WI) than to the South American groups (i.e., Caribbean CoCA, and Andean CoA). Projection to the second component confirmed that the Tanzanian trees were genetically closer to the West Indian racial controls.

**Figure 3:**
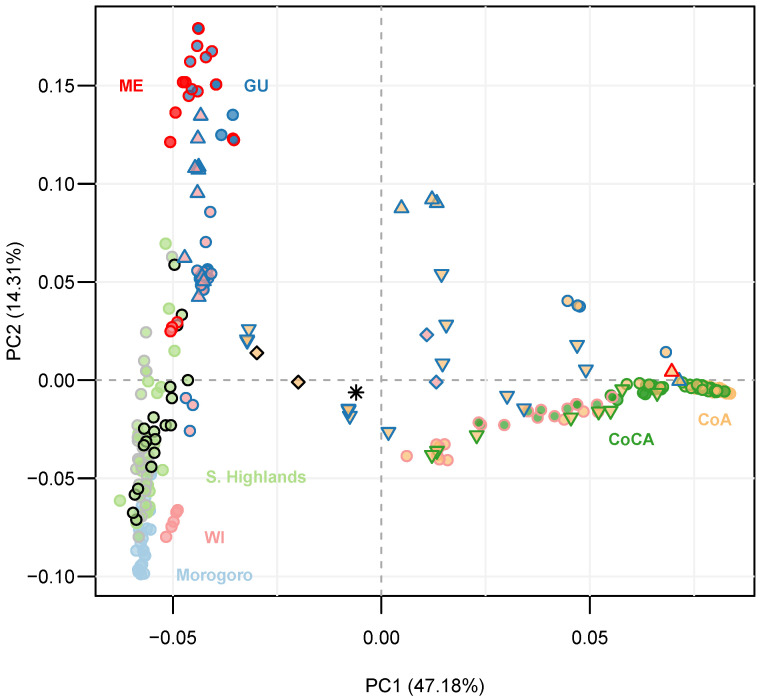
Principal component analysis (PCA) unsupervised genetic clustering of 95 Tanzanian avocado trees from the Morogoro low- to mid-altitude region (light blue) and the Southern Highlands (light green), together with 205 NCBI-available racial controls, all genotyped with lcWGS-derived SNPs. Variance explained by the two main principal components (PCs) is given in the axes’ labels. Trees from the Southern Highlands in Tanzania are discriminated as follows: light green dots with green edges are from the Iringa region, while those with gray and black edges are from the Mbeya and Ruvuma regions, respectively. Racial controls are labeled and colored top down from left to right, following Figure 2: dark red for the Mexican (ME) race, dark blue for the Guatemalan (GU) race, light red for the West Indian (WI) race, and dark green and light orange for the Caribbean (CoCA) and Andean (CoA) groups from northwest South America. The remaining dots mark double hybrids between these last five groups—the interior and the edges are colored according to the putative parental groups. Triangles mark tripe hybrids, with the interior and the edges colored according to two of the putative parental groups, and its direction pointing towards the third contributing group. Diamonds mark tetra hybrids, with the interior and the edges colored according to the two most distant putative parental groups, while the other two groups can be resolved without ambiguity based on the proximity to the five racial control groups. Finally, the asterisk marks the penta hybrid among all five main groups (i.e., Mexican race, Guatemalan race, West Indian race, and Caribbean and Andean groups from northwest South America).

Specifically, the trees from Morogoro were almost exclusively of the West Indian type, while some trees from the Southern Highlands aligned more closely with West Indian × Guatemalan and West Indian × Mexican hybrids (Figure 3). Within the Southern Highlands, trees from the regions of Iringa, Mbeya and Ruvuma were panmictic.

These population stratification patterns were replicable when exploring the high-coverage dataset of 10,460 SNPs (Figure S3). Finally, exploring the third main component against the first (Figure S4) and the second (Figure S5) did not offer further discrimination.

**Figure 4:**
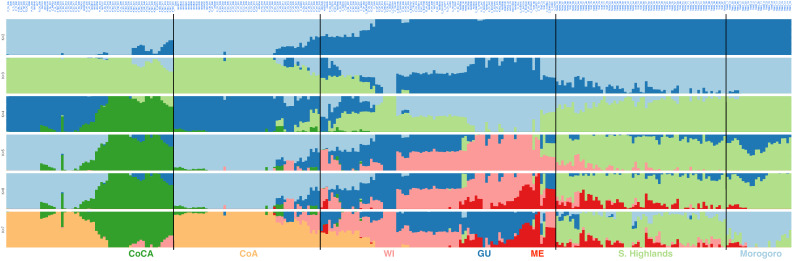
Admixture-based unsupervised genetic clustering (from *K* = 2 to *K* = 7) in 95 Tanzanian avocado tree samples from the low- to mid-altitude Morogoro region and the Southern Highlands, together with 205 NCBI-available racial controls [14], all genotyped with lcWGS-derived SNP markers. Linages at *K* = 7 are colored, from right to left, as in Figure 2: light blue for trees from Morogoro, light green for trees from the Southern Highlands, light red for the West Indian (WI) race, dark red for the Mexican (ME) race, dark blue for the Guatemalan (GU) race, and light orange and dark green for northwest South American Andean (CoA) and Caribbean (CoCA) groups.

ADMIXTURE analysis showed the lowest cross-validation error at *K* = 7 (Figure S6), reinforcing the presence of seven genetic ancestries in the joint panel of 300 trees (Figure 4), and without *K* > 7 (Figure S7) offering additional discrimination. The hierarchy of ancestry segmentation from *K* = 2 to *K* = 7 reflected the patterns of genetic differentiation captured by the previous two main principal components. Specifically, at *K* = 2, the major distinction was between the Mesoamerican races (including the sampled trees from Tanzania) and the two northwest South American control groups. Within the former Mesoamerican cluster, the Mexican and Guatemalan races became distinct from the West Indian race and Tanzanian provenances at *K* = 3, while the latter South American cluster split between the Andean and Caribbean groups at *K* = 4. It was not until *K* = 5 that the Tanzanian provenances detached from the West Indian race, and at *K* = 6 that the disjunction between the Mexican and Guatemalan races was evident. Finally, at *K* = 7, the Tanzanian avocado trees from the Morogoro region and those from the Southern Highlands (i.e., Iringa, Mbeya and Ruvuma regions) separated. Most trees from Tanzania showed high ancestry proportions (>80%) to a single group, as did trees from northwest South America, while the Mesoamerican races exhibited more introgression (Table S8).

## 3. Discussion

The whole-genome analysis presented here unveils for the first time avocado’s diversity and population structure in Tanzania. Tanzanian trees consistently group closer to the West Indian race than to the Mexican or Guatemalan races. Subtle divergence is observed between trees from the low- to mid-altitude Morogoro region and the Southern Highlands, with some West Indian × Guatemalan and West Indian × Mexican introgression in the latter. PCA concordance between the full lcWGS dataset and the more stringent 10,460 high-coverage SNP panel reinforces these inferences. These results offer a whole-genome demographic background landscape against which further evolutionary, adaptative, and breeding signatures may be interpreted to boost local avocados.

### 3.1. Tanzania as a Secondary Center of Avocado Diversification

This study offers the first genome-wide characterization of avocado germplasm from Tanzania and reveals that populations from the Morogoro region and the Southern Highlands form a predominantly West Indian-derived genepool. The strong West Indian ancestry assignment (>80% in most trees) for the Tanzanian samples further suggests relatively recent population expansion from a narrow founder base population [38], rather than deep, long-term admixture across all major racial groups [39]. Nevertheless, the fact that we were also able to capture subtle genetic divergence between trees from the low- to mid-altitudes in Morogoro and those from the Southern Highlands suggests that Tanzania may be regarded as an emerging secondary center of diversification largely shaped by limited Mesoamerican introductions followed by extensive propagation and *in situ* adaptation to the highly heterogenous local topographic and climatic conditions [40].

Furthermore, as part of this diversification pattern, detectable incipient introgression with the other two Mesoamerican races in trees from the Southern Highlands suggests that local environmental and farmer-mediated selection may have indirectly favored segregating Mexican and Guatemalan alleles, already with standing adaptation to cooler mountainous environments. Specifically, trees from Morogoro are almost exclusively West Indian in ancestry, consistent with adaptation to low-elevation, warmer and high-rainfall environments [41,42]. In contrast, the West Indian × Guatemalan and West Indian × Mexican admixture of several trees in the Southern Highlands aligns with the agroecology of these environments, characterized by higher altitudes and cooler temperatures, conditions more similar to those in which the Guatemalan and Mexican races evolved [43]. Also, trees within the Southern Highlands appear largely panmictic across the Iringa, Mbeya and Ruvuma regions, confirming rampant gene flow across this highland agroecological zone rather than strong isolation at the regional level [16,35].

A non-mutually exclusive alternative to ecological and farmer-driven selection favoring introgressed genotypes better suited to cooler environments in the Southern Highlands is additional introductions of non-West Indian provenances. Mesoamerican introductions of fewer non-West Indian trees could have been followed by *in situ* hybridization and introgression [17]. However, at the moment, this ad hoc hypothesis may seem less parsimonious to make sense of the introgression observed in the Southern Highlands, given that no relatively pure Mexican or Guatemalan types were detected in the sampled areas. As a secondary step in the near future, extending the sampling efforts across other regions in Tanzania will help in assessing to what extend the observed regional differentiation reflects ecological filtering or historical re-introduction patterns.

In any case, from an evolutionary perspective, the patterns of allelic filtering and local introgression are both consistent with early-stage divergence in secondary centers, where ecological heterogeneity begins structuring ancestral variants without yet producing deeply differentiated lineages. Such dynamics are particularly relevant for seedling perennial tree crops [10], for which long generation times and allogamy continuously recombine and segregate variation [31,44,45], broadening the base for further selections.

### 3.2. Leveraging Avocado Breeding in East Africa

The predominance of West Indian ancestry in the studied Tanzanian avocado trees impacts varietal selection and rootstock breeding efforts [46,47,48]. West Indian avocados are typically associated with adaptation to lowland torrential tropical climates, higher tolerance to humidity, and larger and more watery fruits [42]. If these provenance’s traits were to be extrapolated, the narrow racial base of avocados from the low- to mid-altitude Morogoro region and the Southern Highlands may have limited standing genetic variation for tolerance to emerging biotic [49,50,51,52,53,54,55,56] and abiotic stresses [57,58], not to mention consumer preferences towards more buttery fruits [36].

Given this bottleneck, the introgressed trees from the Southern Highlands may therefore represent key germplasm for more diversifying breeding [59]. For instance, West Indian × Guatemalan and West Indian × Mexican hybrid backgrounds may depict differential fruit phenotypes and boost adaptation to cooler climates. Such differential fruit phenotypes are supported by Juma et al. [16], who documented four flesh textures, i.e., buttery (58.3%), watery (21.8%), pastose (18.5%) and granular (1.4%), among 226 avocado trees from the Southern Highlands of Tanzania. There may also be additional hidden diversity yet to be disclosed in these populations, for which more exhaustive morpho-agronomic evaluations (as in [16]), extended sampling of additional ‘plus trees’, controlled *ex situ* provenances, progeny and clonal trials (e.g., [22]), and market trade surveys (i.e., [36]) would be highly desirable. Anyhow, the diversity in fruit texture highlights the presence of valuable quality-related traits within Southern Highlands germplasm. Therefore, these genotypes could eventually be utilized as novel clonal varieties by themselves, or alternatively as exotic donor candidates for pre-breeding pipelines, assisted by marker-guided or even genomic-enabled backcross selection schemes [34,60,61], aimed at introgressing differential fruit quality traits and climate change resilience [62].

Similarly, the fact that none of the Tanzanian samples clustered with the Andean or Caribbean groups indicates limited provenance or allelic contribution from northwestern South American germplasm. This recently discovered, yet ancient—originating in the Pleistocene—genepool is starting to be recognized as a cryptic pocket of avocado agrobiodiversity, more genetically distinctive than any Mesoamerican race [14,15]. Since Tanzanian avocado diversification in the studied regions has primarily occurred within the Mesoamerican racial genepool, with no introgression of South American alleles, infusion of elite trees from northwest South America in East Africa may therefore source field trials for rootstock section [63,64,65], and even boost the discovery of promising non-Hass like varieties for particular climates [58,66,67] and market niches [36].

In smallholder systems, farmer preferences and seed exchange networks often act as strong human-mediated selection forces [68]. Given that most trees sampled were ‘plus trees’, maintained and selected by local tribes for decades, it is plausible that unconscious or deliberate selection for fruit size, oil content, taste, or phenology [69] has already shaped allele frequency distributions. This dynamic can generate, over time, locally adapted landrace-like populations even in the absence of formal breeding programs [70]. The relatively high racial ancestry purity observed in many Tanzanian trees may therefore reflect both founder effects, as discussed in the previous section, or even directional and background selection matching local community market demands [36,71,72,73].

Meanwhile, the filtering of a 10,460 high-coverage SNP subset offers a more scalable marker panel for targeted applications [74], such as association mapping of key morpho-agronomic traits [75,76], genomic selection of saplings at the nursery stage [77,78,79], and germplasm diversity management [80,81]. For instance, once markers are escalated to larger training populations, the accuracy of prediction models for complex polygenic traits will increase [82,83], accelerating early indirect selection in nurseries for seedling orchards or as seedling rootstocks for clonal plantations [84].

As global avocado production increasingly relies on a narrower repertoire of commercial cultivars—notably var. Hass—safeguarding regionally adapted germplasm becomes paramount [85]. Secondary diversity centers such as Tanzania are likely to harbor novel allelic combinations conferring elite phenotypes as well as tolerances to yet unforeseen stresses in the region. The lcWGS screening made available here is the first step to characterizing and utilizing this understudied emerging germplasm. Such genomic resources will enable *in situ* conservation efforts, identifying promising as well as underrepresented racial provenances, and guiding hybrid and introgressive breeding.

### 3.3. Perspectives

Future work must envision extending the sampling effort across other Tanzanian regions and neighboring countries in order to improve our understanding of gene flow and diversification dynamics in the secondary diversity center of East Africa. Expanding the base population across local climatic gradients would in turn enable environmental association analyses capable of reconstructing the genomic bases of local adaptation [86], clarifying whether the observed regional stratification corresponds to adaptive divergent selection [87] rather than neutral demographic drift [88] and linage sorting [89]. Controlled provenance and progeny trials would also assist early forward selection of the identified ‘plus trees’ for yield [90], fruit quality [91,92], pathogen resistance, and adaptive traits. Last, moving beyond lcWGS, *de novo* depth-coverage sequencing will eventually allow for pangenomic reconstructions [93,94,95].

## 4. Materials and Methods

### 4.1. Tree Sampling

A total of 95 avocado ‘plus trees’ were sampled in early 2024 across the regions of Morogoro (Mvomero district: 498–1001 m a.s.l., *n* = 25), representing low- to mid-altitude conditions of eastern Tanzania, and Iringa (Iringa district: 1537–1715 m a.s.l., *n* = 20), Mbeya (Rungwe district: 931–1245 m a.s.l., *n* = 30) and Ruvuma (Madaba district: 1219–1450 m a.s.l., *n* = 20) in the Southern Highlands (Table S1). The overall sampling spanned a total four regions, four districts, and seven wards (Table 1).

Six to eight fresh young leaves were collected from each tree, along with GPS coordinates and key tree dasometric measurements—such as tree height and trunk diameter. The leaves were then placed in porous tea bags (three to four leaves per bag) that were labeled with the corresponding sample ID. Thereafter, the bags were stored in plastic boxes containing moisture-absorbing color-indicating silica gel (Sigma Aldrich, Hamburg, Germany) for drying the leaves. Silica gel was replaced until the leaf samples were totally dry. All samples were collected in duplicate is separate boxes.

### 4.2. Genomic DNA Extraction and Low-Coverage Whole-Genome Sequencing (lcWGS)

Genomic DNA was extracted from the 95 silica-dried avocado leaf samples as per the DNeasy Plant Mini Kit (QIAGEN, Hilden, Germany) protocol. DNA was verified in Agarose gels and through the OD260/OD280 ratio (1.8–2.0, >0.5 µg) using a NanoDrop^®^ 2000 spectrophotometer (Thermo Fisher, Waltham, MA, USA). Genomic libraries were prepared using the Illumina TruSeq™ Nano DNA Sample Prep Kit, and sequenced 150 bp pair-end in Illumina Novaseq at CD Genomics (Shirley, NJ, USA).

### 4.3. Sequencing Data Proccessing

Base calling with CASAVA software *v.*1.6 (Illumina Inc., San Diego, CA, USA) was performed to migrate sequencing data recorded in image files to sequence reads. Sequencing quality information was stored in FASTQ files, from which 3′ adapter contamination and low-quality reads were removed using Fastp *v.*0.20.0. Specifically, a 5 bp 3′-5′ sliding window was used to retrieve average *Q* scores. Bases within the window were removed for average *Q* scores < 20. Additionally, paired-end sequences were discarded if their length was <50 bp or the number of N bases was ≥5. Additionally, in order to guide racial ancestry assignment for the Tanzanian trees, FASTQ files from 205 avocados, including 42 commercial varieties, were retrieved from NCBI BioProject ID PRJNA1311704 [14].

Pre-processed data were mapped to the haplotype-resolved Queensland’s avocado cv. Hass reference genome assembly (publicly available in the AvoBase repository: https://www.avocado.uma.es/easy_gdb/downloads.php, accessed on 23 March 2026) [30] using BWA *v.*0.7.12-r1039. Resultant SAM files were transformed to BAM files using Picard *v.*1.107 software (http://www.psc.edu/index.php/user-resources/software/picard, accessed on 23 March 2026) keeping paired-end reads’ consistency with command *FixMateInformation*, and removing duplicates with the *MarkDuplicates* function.

GATK software *v.*4.5.0.0 was used for SNP calling [96]. Variant sites were called using the *Haplotype Caller* function. The resultant GVCF files per sample were merged by a chromosome with the *Combine GVCFs* function, and later combined across chromosomes with the *GatherVcfs* function. SNP markers were extracted from the integrated GVCF file with all samples and chromosomes using the *Select Variants* function, and quality filtering was performed with the *Variant Filtration* function. Specifically, SNPs were retained if Fisher’s test of strand bias (FS) ≤ 60, mapping quality (MQ) ≥ 40, quality depth (QD) ≥ 2, ReadPosRankSum ≥ −8.0, and MQRankSum > −12.5. Retained SNP markers were annotated using ANNOVAR software *v*.4.0 [97]. Finally, a more depurated SNP dataset with higher coverage was obtained using VCFtools software *v.*2015 [98], only keeping markers with a minimum of 10× of sequencing depth (SD) per sample per position, missing data per variant not exceeding 20% (i.e., variants with genotypes present in at least 80% of the samples), and a minimum allele frequency (*maf*) of 5%.

### 4.4. Population Genomics Analysis

To infer the racial ancestry of the 95 Tanzanian samples from Morogoro and the Southern Highlands, phylogenies were built using the full and high-coverage SNP dataset with the 205 published avocado racial controls. Trees were reconstructed using Maximum Likelihood with Shimodaira–Hasegawa node support in Geneious *v.*2026.0.2 [99] FastTree plugin (http://www.microbesonline.org/fasttree/, accessed on 23 March 2026).

Two complementary unsupervised genetic clustering strategies were implemented to confirm the racial ancestry of the 95 Tanzanian samples by relying on the NCBI-available lcWGS data from 205 trees with reported race assignation. First, principal component analysis (PCA) was carried out, retaining three orthogonal dimensions, using GCTA software *v*.1.93.3 (https://github.com/jianyangqt/gcta, accessed on 23 March 2026). This analysis was carried out in the full panel as well as in the high-coverage SNP dataset, inspecting for each case two of the three principal components (PCs) at a time up to the third dimension for the full dataset (i.e., PC1 vs. PC2, PC1 vs. PC3, and PC2 vs. PC3). Second, admixture genetic structure was analyzed using ADMIXTURE software *v*.1.3.1 (http://dalexander.github.io/admixture, accessed on 23 March 2026) by assuming *K* = 2 − 10 *a priori* populations. The analysis provided, given a specific *K* value of assumed populations, a stacked bar plot detailing for each bar (individual) the population ancestry proportions. *K* value was optimized as per the profile of cross-validation (CV) error scores.

## 5. Conclusions

The lcWGS-screened Tanzanian avocado germplasm from the Morogoro region and the Southern Highlands constitutes a predominantly West Indian-derived secondary diversity genepool with introgression signatures from the Guatemalan and Mexican races, and emerging local differentiation. Specifically, the Morogoro low- to mid-altitude region had purer West Indian-type trees, whereas the Southern Highlands (i.e., Iringa, Mbeya and Ruvuma regions) harbored admixed genotypes with putative West Indian × Guatemalan and West Indian × Mexican ancestry, potentially adapted to cooler and high-altitude climates. These findings offer the first insights into the evolutionary history of avocado diversification in East Africa, providing an innovative genomic framework to further harness germplasm conservation and breeding efforts in secondary diversity centers.

## Figures and Tables

**Table 1:** A summary of the 95 avocado ‘plus trees’ sampled in the Morogoro region and the Southern Highlands of Tanzania for low-coverage whole-genome resequencing (lcWGS). The table is sorted according to the elevation ranges. Coordinates are listed for each sampled tree under Table S1.

Region	District	Ward	Number of Samples	Elevation Range (m a.s.l.)
Morogoro	Mvomero	Mzumbe	16	498–936
Morogoro	Mvomero	Muhonda	4	504–516
Morogoro	Mvomero	Mgeta	5	995–1001
Mbeya	Rungwe	Masoko	15	931–1145
Mbeya	Rungwe	Lwanga	15	1172–1245
Ruvuma	Madaba	Wino	20	1219–1450
Iringa	Iringa DC	Ifunda	20	1537–1715

## Data Availability

Processed data is already contained within the article and its Supplementary Materials. On the other hand, raw 95 FASTQ files are available through NCBI BioProject ID PRJNA1442766.

## Supplementary Materials

The following supporting information can be downloaded at https://www.mdpi.com/article/10.3390/ijms27073083/s1.
