# Supplementary material for: Genomic Diversity of Avocado in the Morogoro Region and Southern Highlands of Tanzania
Source: Int J Mol Sci. 2026 Mar 28;27(7):3083. doi: 10.3390/ijms27073083 (PMC13073940; doi:10.3390/ijms27073083)
Supplement: Supplementary file 1 [file ijms-27-03083-s001.zip › Supplementary Materials.pdf]

Table S1. Sampling details of 95 Tanzanian avocado tree samples from the Morogoro low- to mid-altitude region and the Southern Highlands (i.e., Iringa, Mbeya and Ruvuma regions).

Table S2. Overall statistics of sequencing data including number of reads and total number of bases (bp).

Table S3. Quality statistics including high-quality reads count (HQ reads), percentage of high-quality reads relative to the total number of original reads (HQ reads%), number of bases in high-quality reads (HQ bases bp), and percentage of bases in high- quality reads relative to the total number of original bases (HQ bases%).

Table S4. Statistics of mapping rate including total number of reads (total reads), number of reads aligned to the reference genome including single-end and paired-end alignments (mapped reads), and percentage of reads aligned to the reference genome relative to the total number of reads (mapping rate).

Table S5. Statistics of coverage at 1×, 4×, 10× and 20× mean minimum depth per sample.

Table S6. Statistics of SNP calling per sample including number of homozygous genotypes consistent with the reference genome (HOM_REF), number of heterozygous genotypes (HET), number of homozygous genotypes inconsistent with the reference genome (HOM_ALT), number of transitions (Ts, substitutions between purines and purines, or pyrimidines and pyrimidines), number of transversions (Tv, substitutions between purines and pyrimidines), and ratio of transitions to transversions (Ts/Tv).

Table S7. Three main principal components from an unsupervised PCA genetic clustering in 95 Tanzanian avocado tree samples with lcWGS-derived SNP markers.

Table S8. Q matrix from an admixture-based unsupervised genetic clustering (K = 7) in 95 avocado tree samples calibrated with the 205 NCBI-available avocado samples with reported racial ancestry.

Figure S1. Rooted phylogeny of 95 Tanzanian avocado trees from the Morogoro region (light blue) and the Southern Highlands—i.e., Iringa, Mbeya and Ruvuma regions (light green), together with 205 NCBI-available racial controls [14], all genotyped with the full lcWGS-derived SNP panel. Racial controls are consistently colored as follows: Mexican (ME) race in red, Guatemalan (GU) race in dark, and West Indian (WI) in light red. The last branch contains the Andean (CoA) and Caribbean (CoCA) clade from northwest South America (Figure S2).

Figure S2. Unrooted FastTree phylogenetic tree, colored for inspection purposes by provenance, based on lcWGS-derived SNPs screened in 95 Tanzanian avocado trees from the Morogoro low- to mid-altitude region and the Southern Highlands (purple branches), together with 205 NCBI-available racial controls, including 42 commercial varieties (blue branches, var. Hass in red) and trees from northwest South America (orange, yellow and green branches for two subsets and for the entire region, respectively).

Figure S3. Principal component analysis (PCA) unsupervised genetic clustering of 95 Tanzanian avocado trees, together with 205 NCBI-available racial controls, for the high-coverage dataset of 10,460 SNPs (coloring pattern as in Figure S1).

Figure S4. Principal component analysis (PCA) emphasizing the first and the third principal components (PC) in 95 Tanzanian avocado trees, together with 205 NCBI-available racial controls, all screened with lcWGS-derived SNP markers (coloring pattern as in Figure S1).

Figure S5. Principal component analysis (PCA) emphasizing the second and the third principal components (PC) in 95 Tanzanian avocado trees, together with 205 NCBI-available racial controls, all screening with lcWGS-derived SNP markers (coloring pattern as in Figure S1).

Figure S6. Cross-validation (CV) error from the admixture-based unsupervised genetic clustering (from $K = 2$ to $K = 10$) in 95 avocado Tanzanian tree samples, together with 205 NCBI-available racial controls, all genotyped with lcWGS-derived SNPs.

Figure S7. Admixture-based unsupervised genetic clustering (from $K = 2$ to $K = 10$) in 95 avocado tree samples from the Morogoro low- to mid-altitude region and the Southern Highlands in Tanzania, together with 205 NCBI-available racial controls, all screened with lcWGS-derived SNP markers.
