# Supplementary figures and images for: Genomic Diversity of Avocado in the Morogoro Region and Southern Highlands of Tanzania
Source: Int J Mol Sci. 2026 Mar 28;27(7):3083. doi: 10.3390/ijms27073083 (PMC13073940; doi:10.3390/ijms27073083)

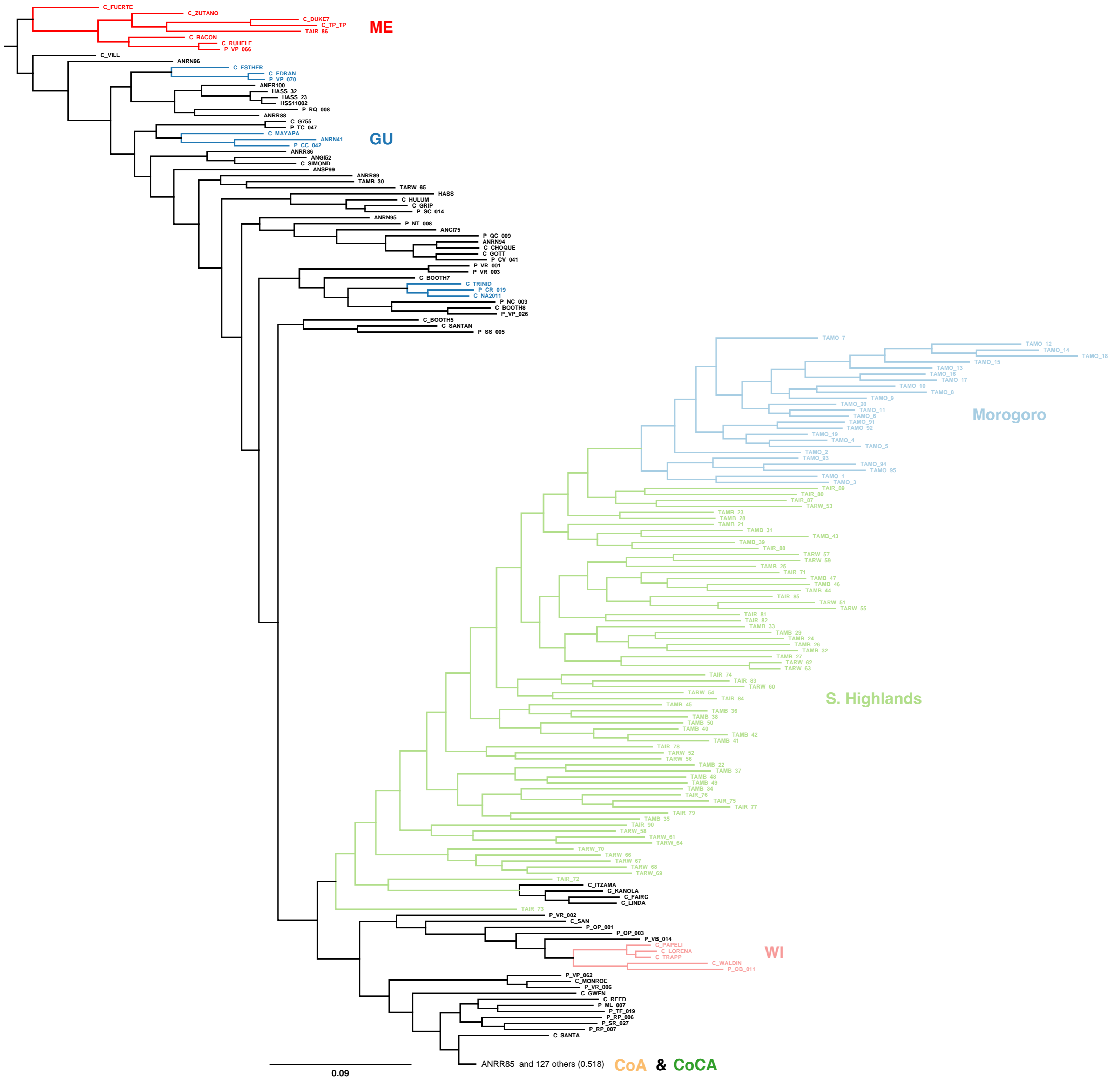

Supplement: Supplementary file 1 [file ijms-27-03083-s001.zip › FigS1.pdf]

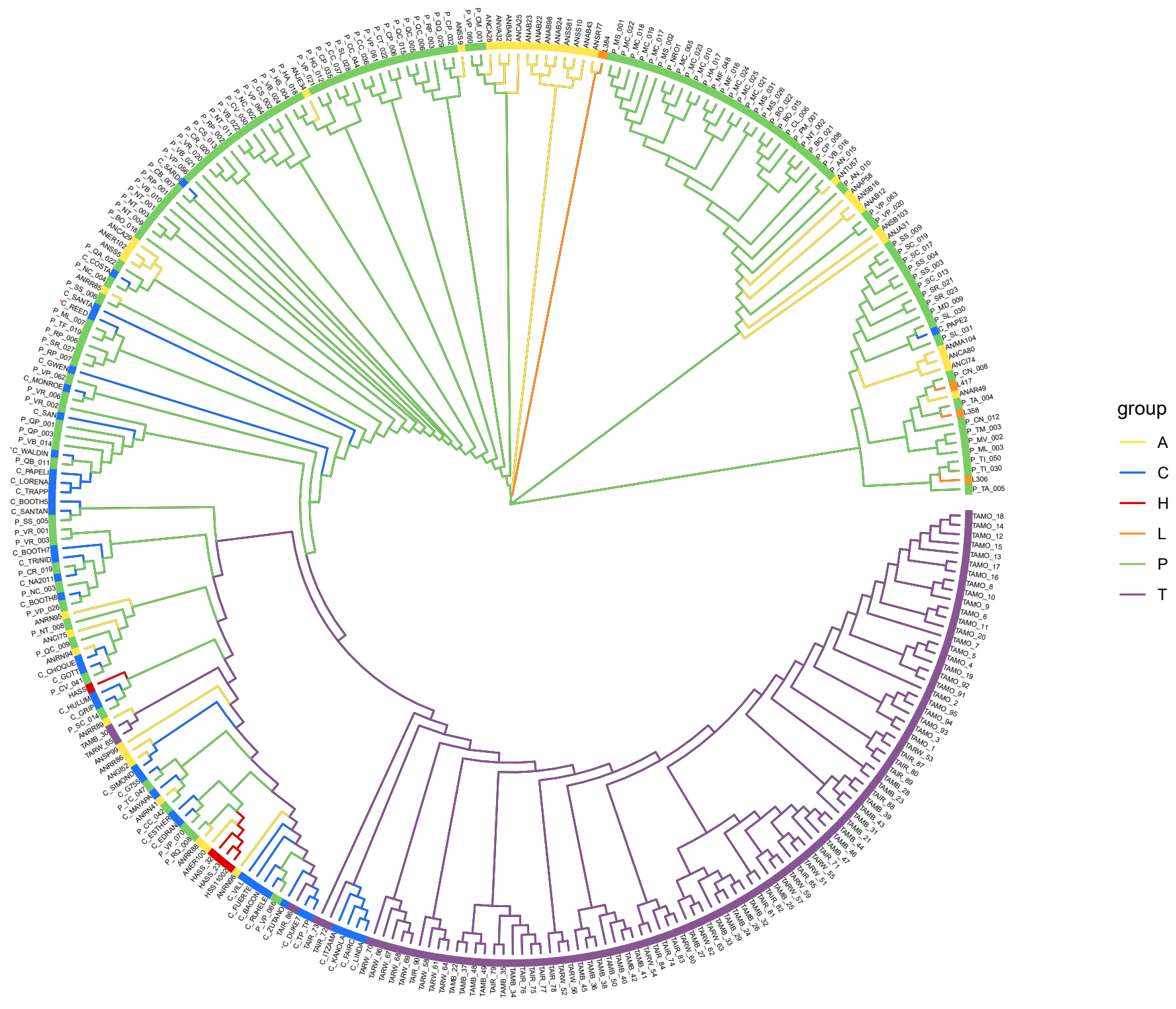

Supplement: Supplementary file 1 [file ijms-27-03083-s001.zip › FigS2.pdf]

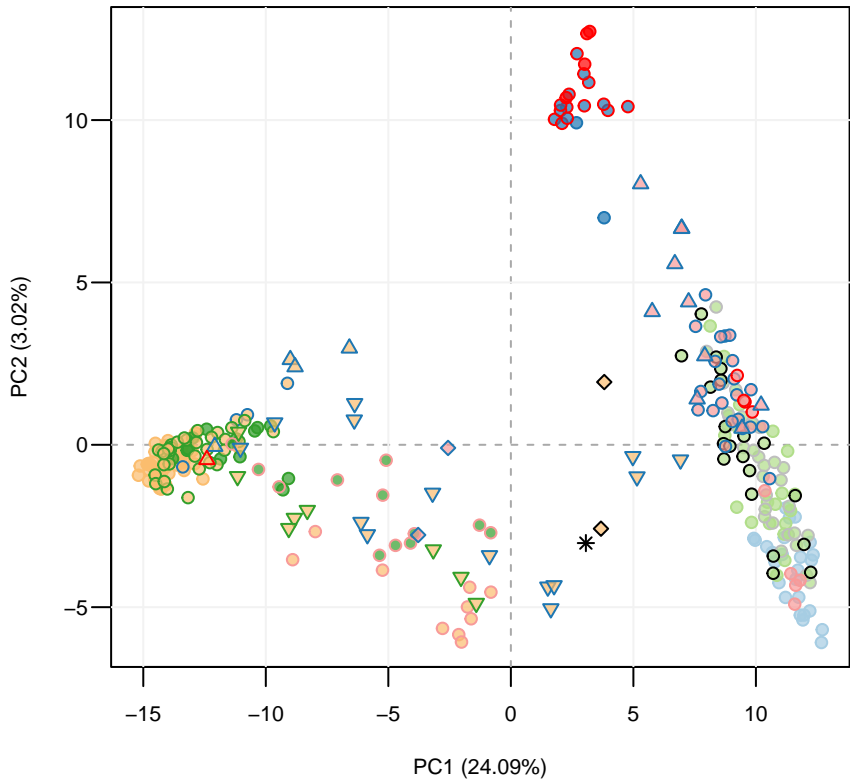

Supplement: Supplementary file 1 [file ijms-27-03083-s001.zip › FigS3_10k.pdf]

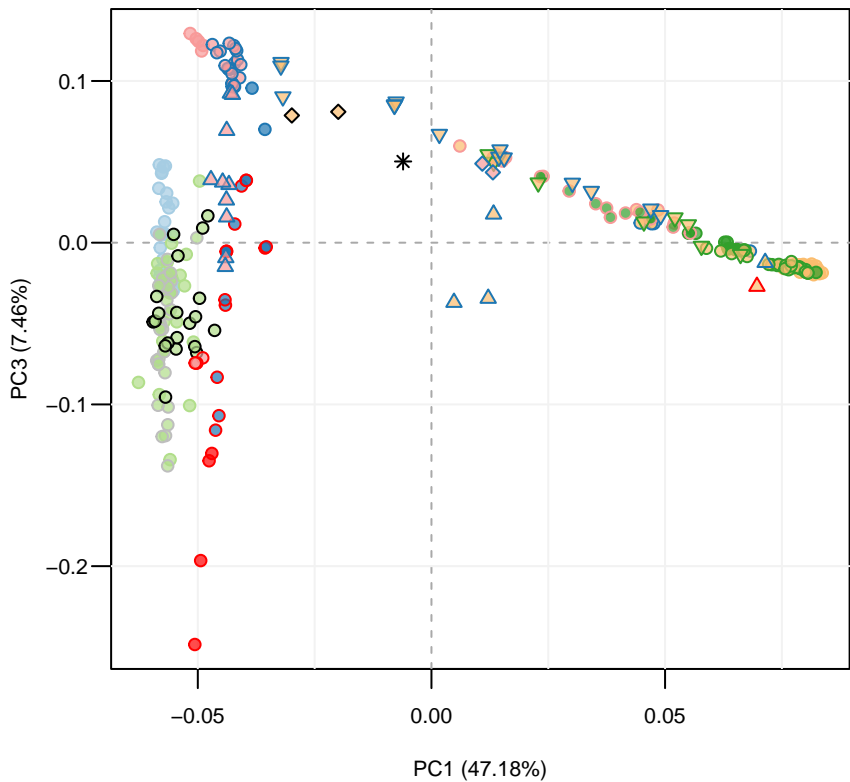

Supplement: Supplementary file 1 [file ijms-27-03083-s001.zip › FigS4.pdf]

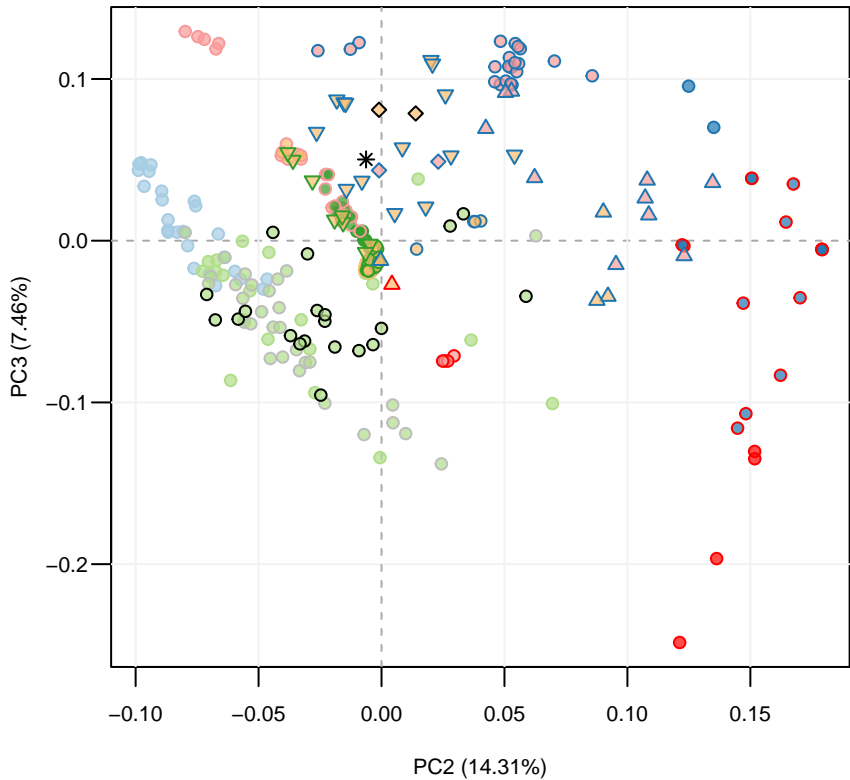

Supplement: Supplementary file 1 [file ijms-27-03083-s001.zip › FigS5.pdf]

Admixture K Estimation

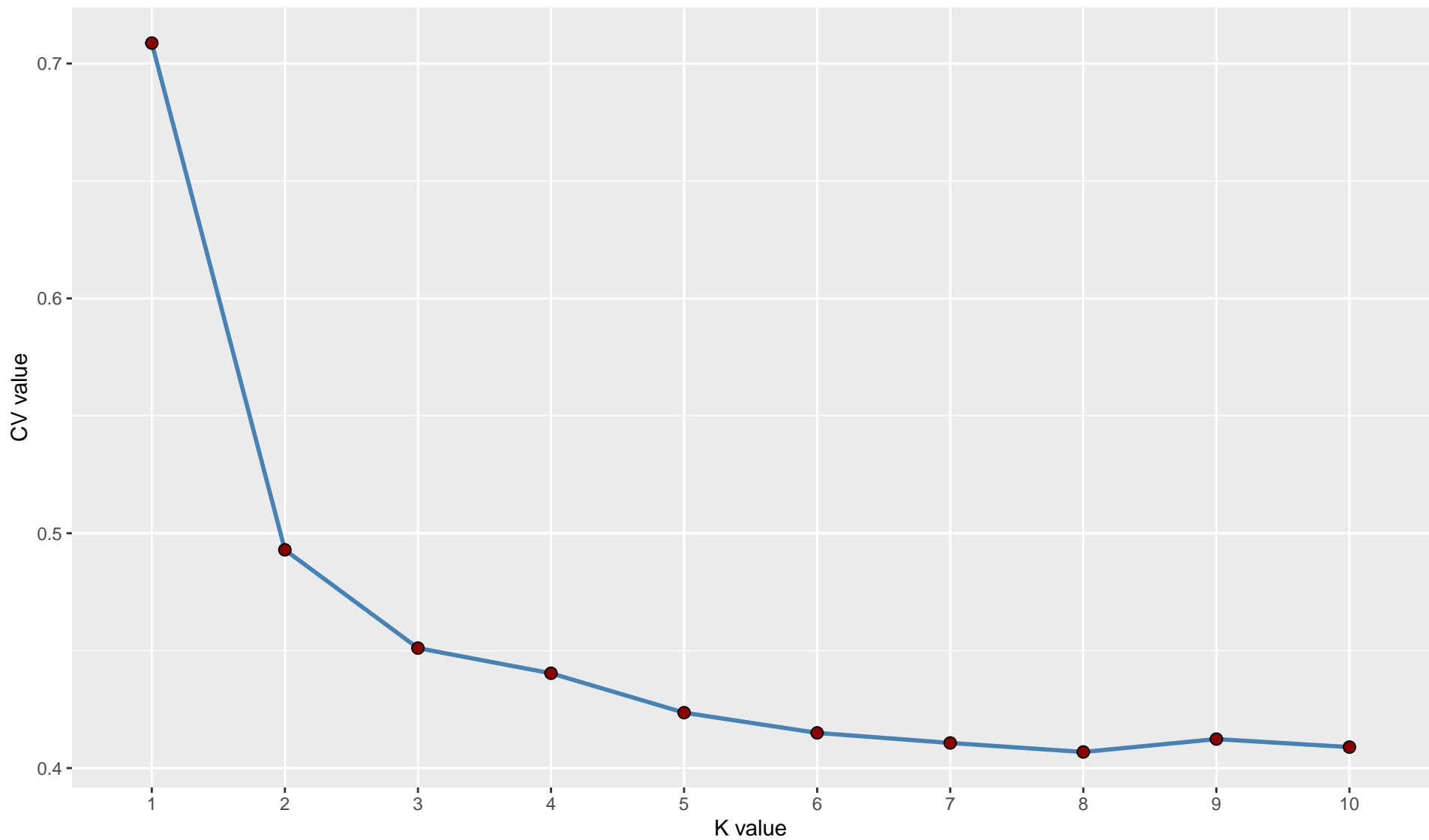

Supplement: Supplementary file 1 [file ijms-27-03083-s001.zip › FigS6.pdf]

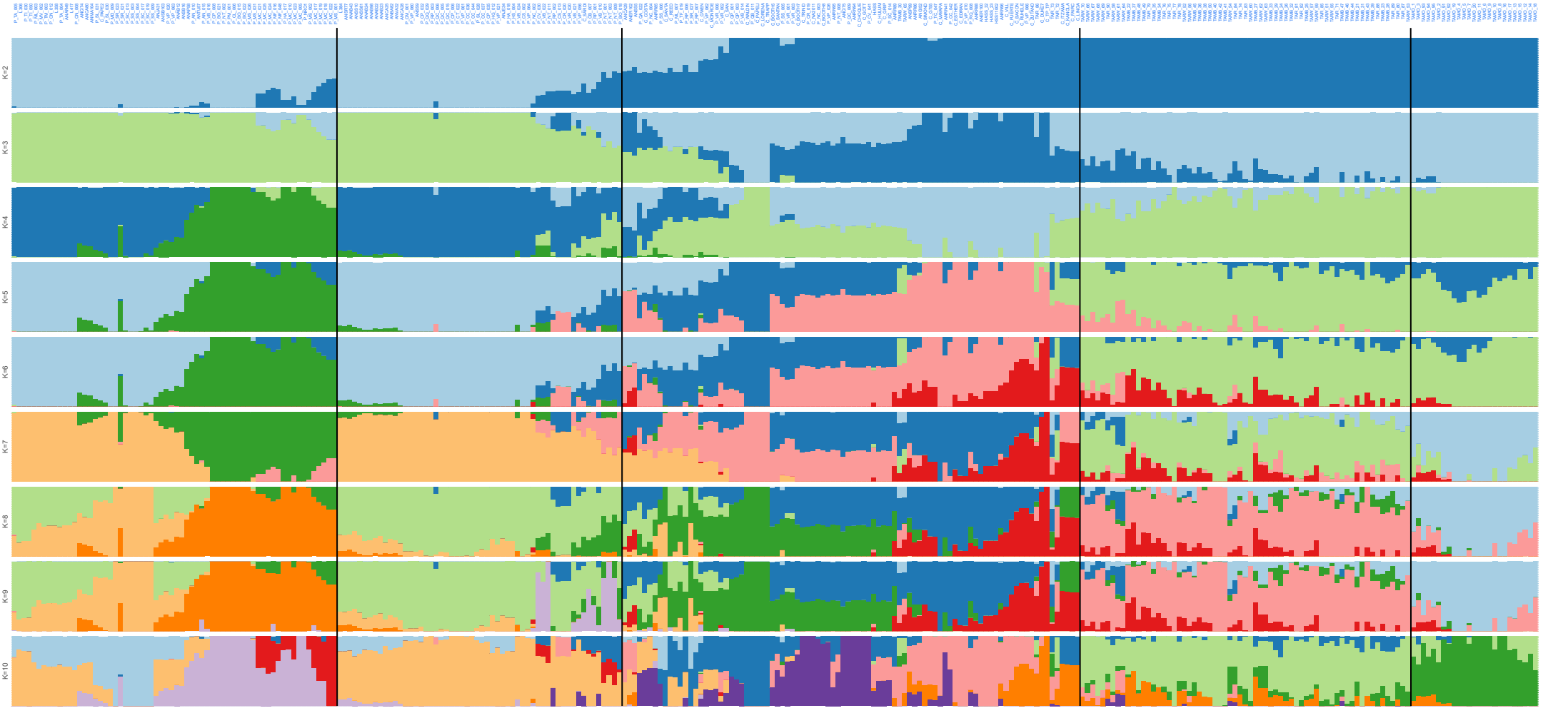

Supplement: Supplementary file 1 [file ijms-27-03083-s001.zip › FigS7.pdf]
